# Redox Homeostasis Disclosed in the Saltmarsh Plant *Halimione portulacoides* upon Short Waterborne Exposure to Inorganic Mercury

**DOI:** 10.3390/toxics12030211

**Published:** 2024-03-12

**Authors:** Patrícia Pereira, Joana Luísa Pereira, Ana Marques, Carlos Marques, Fátima Brandão, Rute Cesário, Silja Frankenbach, João Serôdio, Fernando J. M. Gonçalves, João Canário, Mário Pacheco

**Affiliations:** 1 CESAM and Department of Biology, University of Aveiro, 3810-193 Aveiro, Portugal; pkpereira@ua.pt (P.P.); jpereira@ua.pt (J.L.P.); anammarques@ua.pt (A.M.); carlosamarques@ua.pt (C.M.); fatimabrandao@ua.pt (F.B.); s.frankenbach@ua.pt (S.F.); jserodio@ua.pt (J.S.); fjmg@ua.pt (F.J.M.G.); 2 Centro de Química Estrutural, Institute of Molecular Sciences and Department of Chemical Engineering, Instituto Superior Técnico, Universidade de Lisboa, 1049-001 Lisboa, Portugal; rute.cesario@tecnico.ulisboa.pt (R.C.);

**Keywords:** *Halimione portulacoides*, inorganic mercury, bioaccumulation, antioxidant defenses, oxidative damage, photosynthetic activity

## Abstract

The saltmarsh plant *Halimione portulacoides* was shortly exposed to realistic levels of inorganic mercury (iHg) with the aim of investigating the adaptative processes of the roots and leaves regarding redox homeostasis, physiology, and Hg accumulation. Plants were collected at a contaminated (CONT) and a reference (REF) site to address the interference of contamination backgrounds. The influence of major abiotic variables (i.e., temperature and light) was also examined. Total Hg levels, antioxidant enzymes, lipid peroxidation (LPO), and photosynthetic activity were analyzed after 2 and 4 h of exposure. A poor accumulation of Hg in the roots was noticed, and no translocation to the stems and leaves was found, but plants from the CONT site seemed more prone to iHg uptake (in winter). Despite this, antioxidant modulation in the roots and leaves was found, disclosing, in winter, higher thresholds for the induction of enzymatic antioxidants in CONT leaves compared to REF plants, denoting that the former are better prepared to cope with iHg redox pressure. Consistently, CONT leaves exposed to iHg had remarkably lower LPO levels. Exposure did not impair photosynthetic activity, pinpointing *H. portulacoides*’ ability to cope with iHg toxicity under very-short-term exposure. Biochemical changes were noticed before enhancements in accumulation, reinforcing the relevance of these responses in precociously signaling iHg toxicity.

## 1. Introduction

Saltmarshes are ecosystems of vital importance due to their high biological productivity, hydrological flux regulation, biogeochemical cycling of metals and nutrients, and habitat provision for wildlife [1]. Because of their proximity to major urban centers, these coastal areas commonly serve as sinks for a myriad of contaminants, with potential toxic compounds concentrated in the sediment [2]. As a result, saltmarsh plants are typically exposed to high amounts of trace elements, including mercury (Hg), which has become an environmental and legislative concern due to its widespread prevalence and high toxicity. On the Atlantic coast of Europe, halophytes, such as *Halimione portulacoides*, dominate in saltmarshes in terms of primary production and biomass, while playing important ecological roles in nutrient and contaminant cycling. Previous research indicated a large accumulation of Hg in saltmarsh plants, as well as a significant rise in organic Hg forms in the rhizosphere environment [3]. Recently, it was shown that elemental Hg can be emitted from the leaves of saltmarsh plants, indicating an efficient translocation process inside the plants, and resulting in a low accumulation of Hg in the aerial parts [4,5]. Despite this, there are still many gaps in the understanding of saltmarsh halophytes’ interactions with Hg forms. Considering the pivotal role of these plants in saltmarshes, overcoming these gaps will allow us to predict Hg effects and fate in extremely sensitive ecosystems that are also severely impacted by climate change.

Saltmarsh plants might have developed an ability to cope with the presence of high levels of Hg forms in sediment, namely inorganic mercury (iHg), which is the most abundant Hg counterpart in this matrix [6]. Halophytes inhabiting Hg-contaminated saltmarshes may present specific biochemical and physiological features, likely translating biological attributes to cope with environmental disturbance. Only a few studies have focused on understanding these effects in saltmarsh halophytes under iHg exposure, and most conclusions have been taken from field works. Contrastingly, findings under laboratory-controlled conditions are still scarce and have mainly comprised long-term exposure to iHg, while conclusions provided from a short exposure time window remain elusive. Integrating data from different timescales creates a cohesive narrative about biological effects, as each exposure duration provides a piece of a larger puzzle, elucidating the progression of responses to contaminants and environmental factors, from immediate reactions to potential long-term consequences. Considering all exposure durations provides an accurate and mechanistically based risk assessment, supporting the formulation of effective management strategies. It also helps to identify critical thresholds, vulnerable periods, and potential recovery phases following contamination events. Moreover, when examining the impact of a given environmental factor, the nested nature of exposure durations must be considered, i.e., each larger duration inherently encapsulates smaller time frames. Each level of exposure duration is interconnected, with smaller increments forming the larger units. This interconnectedness implies that observations made within shorter durations contribute to the understanding of the dynamics and effects observed in longer exposures. In light of this perspective, it is evident from the scientific literature analysis that the focus of research on the effects of Hg forms is predominantly directed towards long-term effects, probably due to the recognized features of this metal, like high bioaccumulation potential and persistence. Contrastingly, very-short-term exposures (on a timescale of hours) to Hg have been neglected, devaluating the contribution of immediate responses to uncovering organism’s adaptive mechanisms and to the understanding of broader and longer-term consequences.

Halophytes can tolerate Hg stress via defense mechanisms afforded by the antioxidant system (revised in Natasha et al. [7]). This is because Hg can trigger the production of reactive oxygen species (ROS), which may result in cellular damage, namely protein oxidation, lipid peroxidation, and DNA damage. The role of various enzymatic (e.g., catalase, ascorbate peroxidase, guaiacol peroxidase, and superoxide dismutase) and non-enzymatic (glutathione, phytochelatins, proline, and ascorbic acid) antioxidants in plants has been elucidated with respect to the enhanced generation of ROS and resulting oxidative stress. These key components interact in a sophisticated network, of which the main priority is ROS detoxification and, consequently, the prevention of the cellular injuries referenced above with the pursuit of redox homeostasis. There are several studies linking Hg exposure to the production of ROS in plants (revised in [7]), but only a few were focused on saltmarsh species, and were mostly developed in the field, lacking laboratorial evidence. Recently, a biochemical and lipidomic approach was used to assess the effects of Hg on *H. portulacoides* occurring at two sites of an area historically contaminated with Hg (Laranjo basin, Ria de Aveiro, Portugal), but differing in contamination extent [8]. The enzymatic antioxidant mechanisms protecting membranes [glutathione peroxidase, glutathione S-transferases (GSTs), and dehydroascorbate redutase] were not induced in any of the three analyzed organs (leaves, stems, and roots) [8]. Differently, a decrease in GST activity was reported in the roots and leaves of *H. portulacoides* from the same ecosystem in line with Hg exposure levels [9]. It is predicted that the responses of *H. portulacoides* to Hg exposure could vary depending on environmental temperature or other abiotic factors, in line with what has been recorded in other plants [10], and according to a seasonal fluctuation in accumulated levels [11]. Despite this, no works are available on saltmarsh plants exploring the influence of abiotic factors, such as temperature and light intensity, on biological responses to Hg.

Although saltmarsh halophytes can tolerate trace metal contamination to some extent, excessive levels internalized in the plants can result in the severe impairment of fundamental processes related to protein and energy metabolism [12]. Both antagonistic and synergistic effects between Hg(II) and salinity were confirmed by differential levels of proteins (magnesium chelatase and ribulose-l,5-bisphosphate carboxylase/oxygenase) and metabolites (valine, malonate, asparagine, glycine, fructose, and glucose) in the halophyte *Suaeda salsa* [12]. Moreover, metal overload has been proven to cause serious damage in photosystem II (PS II) [13,14]. Photobiology parameters, such as efficiency and photoprotection capability, were assessed in leaves of *H. portulacoides* exposed to Hg forms [5]. Few differences between the control and exposed plants were observed, indicating the high tolerance of this saltmarsh plant to Hg forms [5]. Pulse Modulated Amplitude (PAM) fluorescence examines photonic energy capture mechanisms and the transformation to electrical energy. Thus, any disturbance at the primary productivity level can be efficiently assessed by this technique.

There are still many questions that need to be clarified associated with the biological attributes of saltmarsh plants, namely *H. portulacoides*, to cope with iHg. Hence, the current research aims to fulfil major knowledge gaps related to the biochemical effects of iHg in a short time exposure window (2 and 4 h) and disclose adaptative responses specific to the roots and leaves regarding redox homeostasis, together with physiological impairments and Hg bioaccumulation. The influence of major abiotic parameters (such as temperature and light) on those responses was also investigated, as well the possible role of the plant’s historic contamination background. Thus, a short-timescale study was carried out in hydroponic conditions, relying on the exposure of *H. portulacoides* to a realistic level of iHg and combining the assessment of root iHg uptake and partitioning with the evaluation of oxidative stress responses to iHg, keeping redox homeostasis and photosynthetic efficiency in mind. This is a powerful approach for the elucidation of *H. portulacoides* plasticity in the presence of a specific environmental disturbance, as well as the way this species is currently contributing to the iHg cycling.

## 2. Materials and Methods

### 2.1. Collection Sites of H. portulacoides

*Halimione portulacoides* species was selected due to its high accumulation propensity to Hg [15]. Plants were obtained in December 2019 and June 2021 to perform, respectively, the winter and the summer experiments (see below Section 2.3). Briefly, plants were collected, during low tide, from a young salt marsh with narrow mudflats, located in the Aveiro lagoon (Murtosa, Portugal). This system has an inner bay (Laranjo bay) that received a highly contaminated effluent between the 1950s and 1994 that was discharged by a mercury cell chlor-alkali plant. In 2005, the Hg levels in the surface sediments of the area ranged from 2.5 to 51.7 μg g^−1^ [16]. Recently, in 2021, total Hg levels in the surface sediments of Aveiro lagoon were determined by the research team of the current study, as part of a broader project, finding out levels comparable to those previously reported in Laranjo basin (22.1 µg g^−1^) [17]. With this in mind, two sampling sites were selected according to relative mercury contamination: a historically contaminated site (CONT; Laranjo basin 40°43′43.3974″ N, 8°36′47.5286″ W) and a minimally contaminated site (Bico 40°42′58.169″ N, 8°39′51.0836″ W), adopted as the reference site (REF; [18]). The levels of other trace elements in the sediment (besides Hg) of the contaminated site were not analyzed in the context of this study. However, this ecosystem is mainly impacted by Hg, making it a field laboratory to support research related to many aspects of this trace element [6].

Laranjo basin has a saltmarsh area where *H. portulacoides* plays an important role in floristic coverage throughout the year. The Laranjo saltmarsh is inundated by tidal action twice a day, which contributes to high detritus exportation to the main system [18]. Plants of similar size were carefully removed from the sediment to ensure that the root system was preserved as much as possible, and were quickly transported to the laboratory.

### 2.2. Chemicals

Hoagland’s modified basal salt mixture was supplied by VWR International, LLC (Radnor, PA, USA). Mercury(II) chloride was purchased from Sigma-Aldrich Chemical Company (St. Louis, MO, USA). All other chemicals were supplied by Sigma-Aldrich Chemical Company (St. Louis, MO, USA) or VWR International, LLC (Radnor, PA, USA).

### 2.3. Experimental Set-Up and Sampling

In the laboratory, *H. portulacoides* specimens were carefully washed with distilled water to remove the adhered sediment particles and placed in a phytoclimatic chamber in 50 mL tubes with ¼ hydroponic Hoagland’s medium [19].

**Figure 1:**
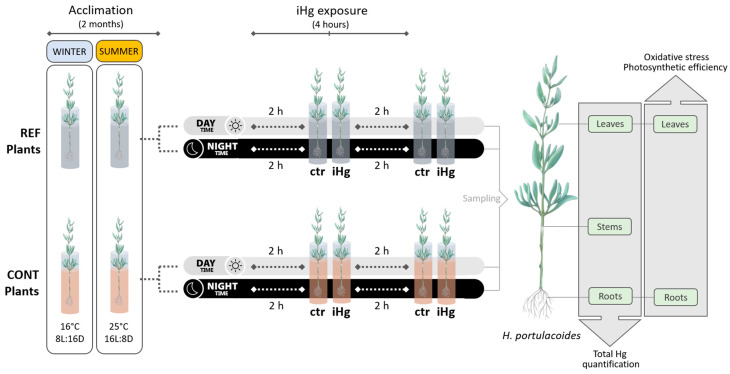
Schematic representation of the experimental design. *Halimione portulacoides* specimens from two different estuarine sites were considered both in winter and summer, namely from a minimally contaminated site (reference; REF) and one historically contaminated with Hg (CONT). Plants of both provenances were hydroponically exposed to 0.45 µg L^−1^ of inorganic Hg (iHg) as Hg(II) chloride for 2 and 4 h (h), within diurnal and nocturnal periods.

Two short-term independent experiments were carried out addressing contrasting seasons, viz. winter and summer (Figure 1). In both experiments, plants were maintained for two months (acclimation period) to allow new root biomass growth, to ensure that plants were in a stable and consistent physiological state before exposure, and to reset Hg levels, if present, due to the field exposure. Plants were kept at a temperature circa 16 °C with an 8:16 (L:D) photoperiod for the winter experiment, and at a temperature circa 25 °C with a 16:8 (L:D) photoperiod for the summer experiment.

Plants were kept under cool white lamps (Phillips Master TL5 HO, 39W/840, Holland) with a PPFD of 115 ± 10 µmol m^−2^ s^−1^. The growth medium was changed every two days throughout the acclimation period.

Following the two-month acclimation, fully developed plants were exposed to 0.45 µg L^−1^ mercury(II) chloride (iHg) for 2 and 4 h, and different conditions of daylight were also simulated (day vs. night) (Figure 1). This experimental variable was considered because whilst most research has been focused on the effects of metals on photosynthesis [20], not much is known on the possible short-term effects of photosynthetic activity on Hg(II) uptake.

The levels of Hg(II) selected for the experiment were based on previous data, namely from field campaigns carried out at the Laranjo site. This study was performed as part of a broader research study which comprised field campaigns in the sampling sites with the main purpose of assessing environmental levels of Hg forms. The total Hg level found in the porewaters of the Laranjo site was 457 ng L^−1^ (Menezes et al., in preparation). Thus, the selected levels of Hg(II) for the experiment with *H. portulacoides* will allow for conclusions to be translated to realistic exposure scenarios.

The experimental trials were conducted in 50 mL RackLock DigiTUBEs (SCP Science, Canada) made of polypropylene with ultralow catalytic/additive metal content under the same temperature and light conditions considered in the acclimation period. Each tube contained one plant, individually, and 40 mL of Hg-spiked Hoagland’s medium. In parallel, the plant control sets were kept in Hoagland’s medium containing only nutrients under the same experimental setting conditions [5]. Three individual replicates for each treatment were attained. At each sampling point, the leaves and roots of each plant were harvested, weighted, washed, and fractionated for the quantification of total Hg levels (tHg) (see Section 2.4), as well as for the assessment of oxidative stress responses (see Section 2.5), and stored at −20 °C and −80 °C, respectively, until further processing. Stems were also sampled for tHg quantification and stored at −20 °C. Five leaves of each plant were also separated to perform the assessment of photosynthetic efficiency.

### 2.4. Quantification of Total Hg in Plant Organs

*Halimione portulacoides* roots, stems, and leaves were oven-dried at 40 °C and homogenized using a mortar. Then, total Hg (tHg) levels were quantified by thermal pyrolysis atomic absorption spectroscopy (LECO AMA 254 system). All samples were analyzed in triplicate. International certified reference materials BCR-60 (aquatic plant, *Lagarosiphon major*) and BCR-61 (aquatic moss, *Platyhypnidium riparioides*) were used to ensure the accuracy of the procedure. Mercury concentrations were consistently within the ranges of the certified values. Blanks were repeated every 20 samples to evaluate cross contaminations and to ensure that the equipment was operating in the same conditions. The limits of detection (LOD) and quantification (LOQ) were calculated as three and ten times the standard deviation from the blanks, respectively. Precision was better than 4.0% (*n* = 40), expressed as a percentage relative standard deviation [21]. The levels of other trace metals in the plants were not analyzed prior to the experiment, but the interference of other elements in the recorded effects should be negligible for the following reasons: (i) the occurrence of other metals besides Hg in the studied areas is low; (ii) the long acclimation period of 2 months should have been enough to reset those small levels in the plants, namely by allowing the growth of new radicular tissues; (iii) the plants were solely exposed to Hg(II).

### 2.5. Oxidative Stress Assessment

Sample preparation for the biochemical analysis was performed based on previously described methods [22], with slight modifications noted in the following subpoints. Parameters related to oxidative stress were not assessed in the stems, since their hardness makes it difficult to complete proper homogenization and extract preparation.

#### 2.5.1. Enzymatic Antioxidants

All enzymatic analyses were performed at 25 °C, according to Duarte et al. [22]. Briefly, the samples were homogenized (ground in liquid nitrogen with a mortar and pestle) and mixed with the extraction buffer [50 mM sodium phosphate buffer at pH 7.6 with 0.1 mM ethylenediaminetetraacetic acid disodium salt dihydrate (Na-EDTA), 1% polyvinylpyrrolidone (PVP), 0.1 mM phenylmethylsulfonyl fluoride (PMSF), and 1 mM dithiothreitol (DTT) (dissolved in the extraction buffer right before the homogenization)] in a ratio of 0.05 g of plant material to 0.8 mL of extraction buffer [22]. Then, the homogenate was centrifuged at 14,000× *g* for 20 min at 4 °C, and the supernatant was frozen at −80 °C until further use for enzymatic analysis.

Catalase (CAT) activity was measured according to a previously described method [23] by monitoring the consumption of H_2_O_2_ and the consequent decrease in absorbance at 240 nm (ε = 43.5 M^−1^ cm^−1^) in a SpectraMax 190 microplate reader. The reaction mixture contained 50 mM of sodium phosphate buffer (pH 7.6), 0.1 mM of Na-EDTA, and 100 mM of H_2_O_2_. The reaction was initiated by adding 10 μL of the extract to 200 μL of the reaction mixture. The results are expressed as μmol H_2_O_2_ min^−1^ mg protein^−1^.

Ascorbate peroxidase (AP) activity was assayed according to Tiryakioglu and colleagues [24]. The reaction mixture contained 50 mM of sodium phosphate buffer (pH 7.6), 12 mM of H_2_O_2_, and 0.25 mM of L-ascorbate. The reaction was initiated with 10 μL of enzymatic extract and 200 μL of reaction mixture. The activity was recorded as the decrease in absorbance at 290 nm in a SpectraMax 190 microplate reader, and the amount of ascorbate oxidized was calculated (ε = 2.8 mM^−1^ cm^−1^). The results are expressed as mol oxidized ascorbate min^−1^ mg protein^−1^.

Guaiacol peroxidase (GP) activity was measured by the adapted method described by Duarte and colleagues [22] using a reaction mixture consisting of 50 mM of sodium phosphate buffer (pH 7.6), 2 mM of H_2_O_2_, and 20 mM of guaiacol. The reaction was initiated with 10 μL of enzymatic extract and 200 μL of reaction mixture. The enzymatic activity was measured by monitoring the increase in absorbance at 470 nm (ε = 26.6 mM^−1^ cm^−1^) in a SpectraMax 190 microplate reader. The results are expressed as mol oxidized guaiacol min^−1^ mg protein^−1^.

Superoxide dismutase (SOD) activity was assayed with a Ransod kit (Randox Laboratories Ltd., UK). The method employs xanthine and xanthine oxidase to generate superoxide radicals which react with 2-(4-iodophenyl)-3-(4-nitrophenol)-5-phenyltetrazolium chloride (INT), forming a red formazan dye. Then, SOD activity is measured by the degree of inhibition of this reaction, considering that one SOD unit causes a 50% inhibition in the INT reduction rate, under the conditions of the assay. The enzymatic activity was measured by monitoring the increase in absorbance at 505 nm in a SpectraMax 190 microplate reader and the results are expressed as SOD units mg protein^−1^.

Total protein was determined according to the Bradford method [25] with the Bio-Rad protein assay kit (Bio-Rad Laboratories Inc., Hercules, CA, USA), using bovine serum albumin (BSA) as the standard protein.

#### 2.5.2. Estimation of Lipid Peroxidation

The content of thiobarbituric acid-reactive substances (TBARSs) was measured adopting previously described methods [22,26,27] with some modifications. Briefly, the leaf and root vegetative material (approx. 0.10 g) were homogenized (ground in liquid nitrogen with a mortar and pestle) and mixed with 1 mL of 0.5% 2-thiobarbituric acid (TBA) and 20% trichloroacetic acid (TCA). The homogenate was heated at 95 °C for 30 min and the reaction was immediately stopped in ice. Then, samples were centrifuged at 3000× *g* for 5 min at 4 °C. The absorbance of the supernatant was read at 532 nm and 600 nm in a SpectraMax 190 microplate reader. The rate of lipid peroxidation was expressed as nanomoles of TBARS formed per gram of fresh weight using a molar extinction coefficient of 1.55 × 10^5^ M^−1^ cm^−1^.

### 2.6. Photosynthetic Efficiency Assessment

Plants were assessed for their responses to Hg exposure regarding photosynthetic activity using Pulse-Amplitude-Modulated (PAM) fluorometry. Briefly, five leaves of each plant were taken and kept in a dark case until the beginning of each analysis. The following photophysiological parameters were nondestructively measured on leaves of the studied species: Fv/Fm and dF/Fm’ (the maximum and effective quantum yields of photosystem II (PSII)) and the rapid light curves of ETR (the relative electron transport rate at PSII, a proxy for photosynthetic rates). An imaging fluorometer (Open FluorCAM, Photon Systems Instruments, Drásov, Czech Republic) was used, allowing imaging fluorometry for the high-throughput analysis of a large number of samples [28].

### 2.7. Statistical Analysis

A two-way ANOVA approach was applied to assess the potential interactive effects of collection site (REF, CONT) and treatment (control, iHg) on the response of each biomarker in the leaves and roots following a 2 h or a 4 h exposure period, during the day or during the night. The same approach was applied to understand the effects of the factors on photosynthetic efficiency and iHg accumulation in the roots, stems, and leaves. The related assumptions of normality and the homogeneity of variances were confirmed using the Shapiro–Wilk and Levene’s tests, respectively. In the few cases where these assumptions were not met even following the application of different data transformation approaches (see footnotes of Tables S1–S3), parametric tests were still run considering that the deviations found were not severe (residual graphical analysis) in order to keep consistency among all analyses. When the interaction was significant, a post hoc pairwise multi-comparison approach was followed using the Tukey test. The assumed alpha level was 0.05 in all analyses.

## 3. Results

### 3.1. Mercury Accumulation

**Figure 2:**
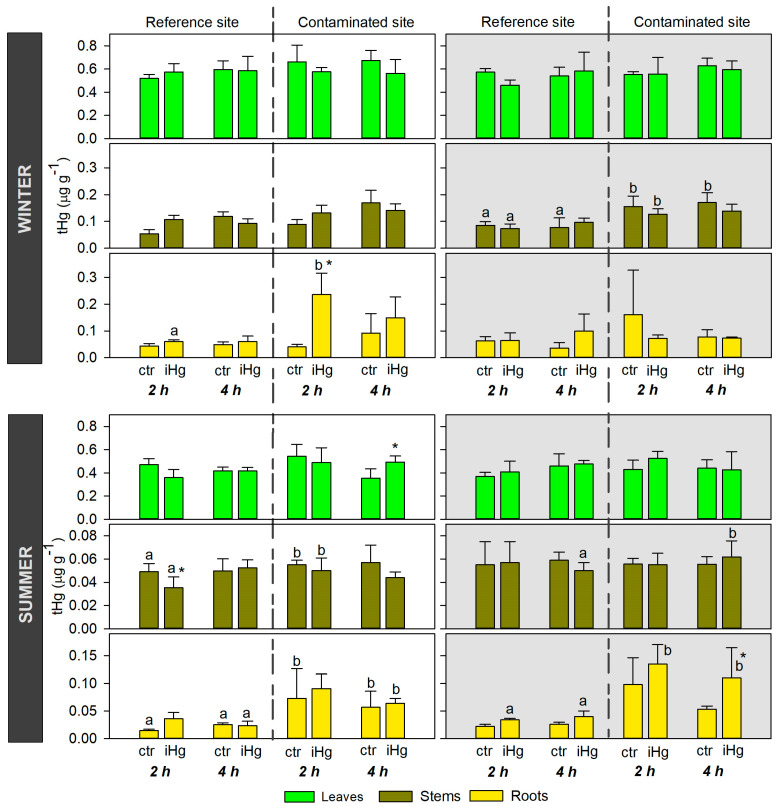
Total mercury (tHg) levels in the roots, stems, and leaves of *Halimione portulacoides* from two different estuarine sites—minimally contaminated (reference) and historically contaminated with Hg—collected in the winter and in the summer, and laboratory-exposed to 0.45 µg L^−1^ inorganic Hg (iHg) for 2 and 4 h during the day (left-hand white panels) or the night (right-hand gray panels). For each plant provenance, values (each stack represents the mean of three replicates) concern unexposed (ctr) and exposed (iHg) groups. Statistical differences are given based on Tukey tests following each omnibus two-way ANOVA, as summarized in Table S1 (*p* < 0.05). * Significantly different from ctr within the same site/origin and exposure length; different letters represent significant differences between different sites/origins within the same treatment.

The statistical differences between treatments (within site/origin and exposure length) were small, but overall, they were more conspicuously recorded in the roots, while a single increase of Hg was found in the leaves upon iHg exposure, and no enhancements were recorded in the stems (Figure 2 and Table S1). A significant enrichment in Hg levels was recorded in the roots of CONT plants immediately after 2 h of exposure in winter-simulated conditions. A slight increase was also recorded after 4 h. However, no significant increases due to exposure were recorded in the stems and leaves of those plants, and no significant changes of Hg levels were found in the roots, stems, or leaves of the plants exposed to iHg under dark conditions in winter.

In the experiment conducted in summer, a single significant enrichment in Hg levels following exposure was recorded in the roots of plants from the CONT site, i.e., upon 4 h of exposure to iHg under darkness. This variation was not followed by significant enrichments in Hg in the stems or leaves. In addition, exposure triggered an accumulation of Hg in the leaves of CONT plants after 4 h of exposure under light conditions.

In winter, the roots of CONT plants had significantly higher Hg levels than those from the REF site upon 2 h of exposure to iHg at daylight (average values of 0.24 and 0.06 µg g^−1^, respectively), while no differences were recorded at control conditions. An identical trend was found for the stems of CONT plants exposed to iHg for 2 h in the dark. However, the stems of plants from the CONT site already had higher levels of Hg than those from REF, regardless of iHg exposure (meaning levels of unexposed plants at 2 h of exposure), namely 0.16 and 0.09 µg g^−1^ at 2 h, respectively. An identical difference was found at 4 h of exposure for the stems of unexposed plants. In general, in summer, the roots of plants from the CONT site showed higher levels of Hg than those from the REF site when exposed to daylight. This pattern was recorded both for control conditions and upon iHg exposure (a single exception was found; although CONT plants exposed to Hg(II) for 2 h had higher levels of Hg than those from the REF site, no statistical differences were found). Interestingly, when the exposure was conducted under darkness in summer, differences related to plant provenance were only recorded upon iHg exposure, with the roots of the CONT plants having higher Hg levels (0.13 and 0.11 µg g^−1^ at 2 and 4 h, respectively) than those from the REF site (0.03 and 0.04 µg g^−1^ at 2 and 4 h, respectively). Contrastingly, the leaves of the plants from distinct provenances had identical Hg levels in control and iHg-exposed conditions, both in winter and summer.

A summary interpretation of Figure 2 highlights that tHg burden in the plants was modulated by season. Regardless of the experimental treatment, plants, mainly those of CONT origin, tended to have more Hg in their tissues in the winter than in the summer. The average values of Hg levels in the three organs of the plants from the CONT site were always above 0.60 µg g^−1^ in winter, while this sum was lower for the summer. The differences in the overall Hg plant burden between exposures held at night and during the day are not apparently remarkable within each season.

### 3.2. Oxidative Stress Endpoints

The analysis of oxidative stress biomarkers was only carried out in the roots and in the leaves, since the processing of stem structures was not feasible due to methodologic drawbacks related to stem hardness that constrained sample preparation and restricted feasible results.

#### 3.2.1. Enzymatic Antioxidant Defense

**Figure 3:**
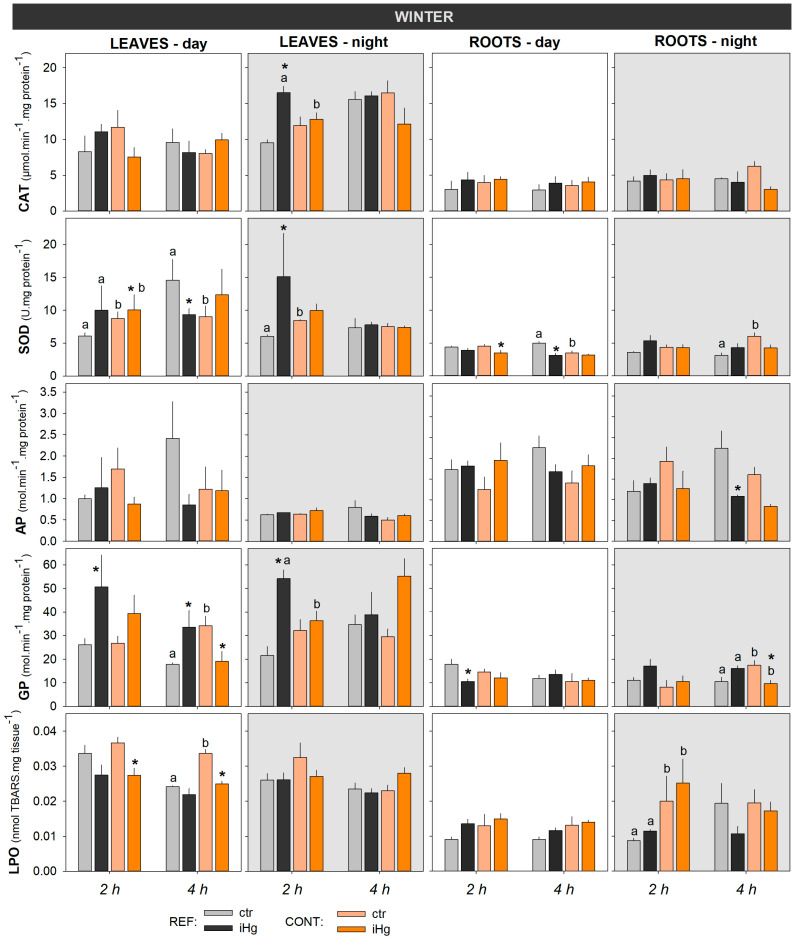
Oxidative stress responses in the leaves and roots of *Halimione portulacoides* collected/exposed in the winter, depicting the activity of catalase (CAT), superoxide dismutase (SOD), ascorbate peroxidase (AP), and guaiacol peroxidase (GP), as well as lipid peroxidation (LPO) levels measured as thiobarbituric acid-reactive substances (TBARSs). Plant specimens were collected from two different estuarine sites—minimally contaminated (reference; REF) and historically contaminated with Hg (CONT)—and laboratory-exposed to 0.45 µg L^−1^ inorganic Hg (iHg) for 2 and 4 h during the day (left-hand white panels) or the night (right-hand gray panels). For each plant organ and provenance, values (bars represent the mean of three replicated measurements and the error bars represent the standard error) concern unexposed (ctr) and exposed (iHg) groups. Diurnal and nocturnal effects of site/origin (REF vs. CONT) and treatment (ctr vs. iHg) in terms of statistical differences are given based on Tukey tests following each omnibus two-way ANOVA, as summarized in Tables S2 and S3 (*p* < 0.05). * Significantly different from ctr within the same site/origin and exposure length; different letters represent significant differences between different sites/origins within the same treatment.

**Figure 4:**
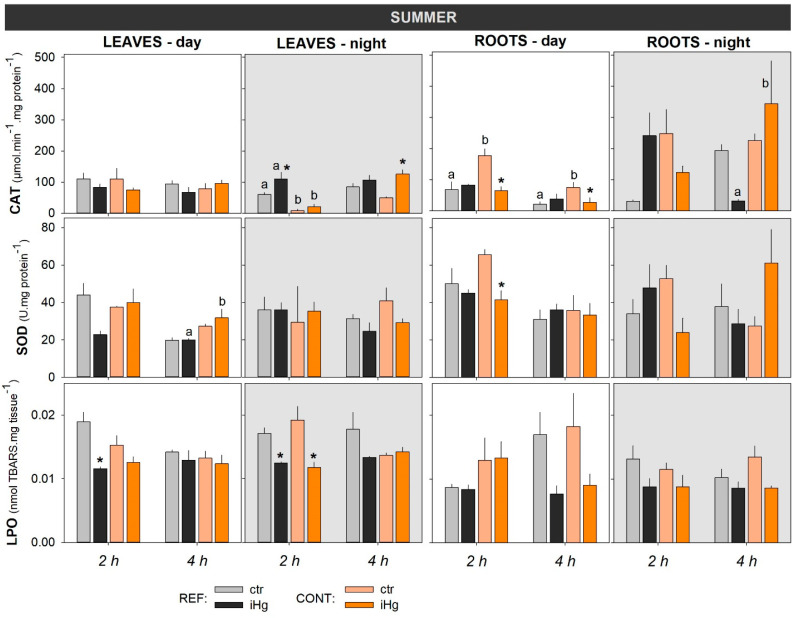
Oxidative stress responses in the leaves and roots of *Halimione portulacoides* collected/exposed in the summer, depicting the activity of catalase (CAT) and superoxide dismutase (SOD), as well as lipid peroxidation (LPO) levels measured as thiobarbituric acid-reactive substances (TBARS). Plant specimens were collected from two different estuarine sites—minimally contaminated (reference; REF) and historically contaminated with Hg (CONT)—and laboratory-exposed to 0.45 µg L^−1^ inorganic Hg (iHg) for 2 and 4 h during the day (left-hand white panels) or the night (right-hand gray panels). For each plant organ and provenance, values (bars represent the mean of three replicated measurements and the error bars represent the standard error) concern unexposed (ctr) and exposed (iHg) groups. Diurnal and nocturnal effects of site/origin (REF vs. CONT) and treatment (ctr vs. iHg) in terms of statistical differences are given based on Tukey tests following each omnibus two-way ANOVA, as summarized in Tables S2 and S3 (*p* < 0.05). * Significantly different from ctr within the same site/origin and exposure length; different letters represent significant differences between different sites/origins within the same treatment. AP activities were undetectable, while GP was not analyzed due to a lack of samples.

Enzymatic antioxidants assessed in leaves and roots evidenced that CAT and SOD activities were differentially affected by season in terms of responsivity to iHg. Hence, CAT activity was more responsive in the summer than in winter, more widely distinguishing exposure to iHg and the plants’ sourcing sites, while the opposite was observed for SOD activity (Figure 3 and Figure 4; Tables S2 and S3). The CAT response to iHg exposure in winter was sporadic, with its activity changing significantly (increasing) only in leaves from plants collected in the REF site following 2 h of nocturnal exposure (Figure 3 and Table S2). In the summer (Figure 4 and Table S3), CAT activity varied more conspicuously, tending to increase significantly in leaves following iHg nocturnal exposure and to decrease in roots following diurnal exposure. There was no clear association between this pattern and the plants’ sourcing sites, but the leaves of plants from the CONT site tended to show lower CAT activity than the those from the REF site, while the opposite seemed to occur for the roots.

As for SOD activity, in the winter, significant changes were noticed following exposure to iHg, yet they were not particularly consistent (Figure 3 and Table S2). Thus, in the leaves, iHg promoted an increase in SOD activity after 2 h of diurnal exposure in the CONT group, while in the REF group a decrease after 4 h of diurnal exposure and an increase after 2 h of nocturnal exposure were detected. In the roots, SOD activity decreased following 2 and 4 h of diurnal exposure to iHg for plants from the CONT and REF groups, respectively. The trends concerning differences in SOD activity between plants’ sourcing sites are also inconsistent. For example, after 2 h of iHg exposure, there was a higher activity of SOD in the leaves from the diurnal control treatment composed of plants from the CONT site compared to the REF site, but the opposite was found after 4 h of exposure (Figure 3, left panel; Table S2).

Both organs were poorly responsive to iHg in summer in terms of SOD activity. A single significant alteration (activity decline) was detected in the roots of plants from the CONT group following 2 h of exposure during daytime. The plants’ provenience significantly affected SOD activity only when the leaves of iHg-exposed groups (4 h) were compared, with the CONT group showing higher levels.

Overall, the activities CAT and SOD were clearly higher in the summer than in the winter regardless of the plant origin, the diel period, or the exposure treatment; on the other hand, there seemed to be a trend for a lower activity of these antioxidant enzymes in the roots than in the leaves in winter, while this was not so clear in the summer (see Figure 3 and Figure 4, comparatively).

Winter data revealed that AP activity was largely unresponsive to iHg (regardless of sourcing site) and their interaction, i.e., a single significant change (decrease), was found when roots from the REF site were exposed for 4 h during the night (Figure 3 and Table S3). In summer, AP activity showed no measurable levels, which is the reason why data are not shown.

Still, in winter, the activity of GP was consistently higher in the leaves than in the roots, while the influence of the diel period could not be clearly ascertained (Figure 3 and Tables S2 and S3). Nonetheless, during both day and night periods, leaves from the REF site were more responsive to iHg; roots sourced from the CONT site were more responsive in experiments held during the night than during the day, a diel period where GP in roots was unresponsive in general. Leaves from the plants sourced from the REF site were consistently responsive to iHg, with GP activity significantly increasing in the treated groups compared to the control (statistics did not confirm the increase only for the 4 h exposure during the night), while in plants from the CONT site, a significant decrease in GP activity was eventually noticed following a 4 h diurnal exposure. In summer, GP was not analyzed due to the lack of samples (accidentally missed), which is the reason why data are missing in Figure 3.

#### 3.2.2. Peroxidative Damage

Regarding LPO, levels in the leaves tended to be higher in winter than in summer, but similar levels were found for the roots in the two seasons. Interestingly, oxidative damage was fully avoided regardless of the season, the iHg treatment involved, and the sourcing site of the plants. When significant changes in LPO levels were found in the leaves after exposure to iHg, these always showed a decrease in the endpoint record (Figure 3 and Figure 4, leaves; Tables S2 and S3). In winter, LPO levels in the leaves were unresponsive to iHg in nocturnal exposure, but after diurnal exposure significantly lower levels were found in plants from the contaminant site after exposure to iHg for 2 and 4 h, yet plants of the REF site were not responsive (Figure 3 and Table S2). In the summer, LPO levels in leaves responded to iHg after diurnal exposure for 2 h but not for 4 h, and the change was only noted in plants sourced from the REF site. The same lack of response was found in LPO levels after 4 h of exposure, while a decrease found after 2 h of exposure in plants sourced from both the REF and CONT sites was recorded in nocturnal exposures (Figure 3 and Table S3). Higher levels of LPO were generally found in plants sourced from the CONT site compared to those sourced from the REF site, particularly in the winter; this difference was statistically confirmed in leaves exposed to iHg for 4 h during the day, as well as in roots from the control and the iHg treatment after 2 h of nocturnal exposure (Figure 3 and Table S2). In general, roots were largely unresponsive to iHg exposure, regardless of season, diel period, or plant origin.

### 3.3. Photosynthetic Efficiency

**Figure 5:**
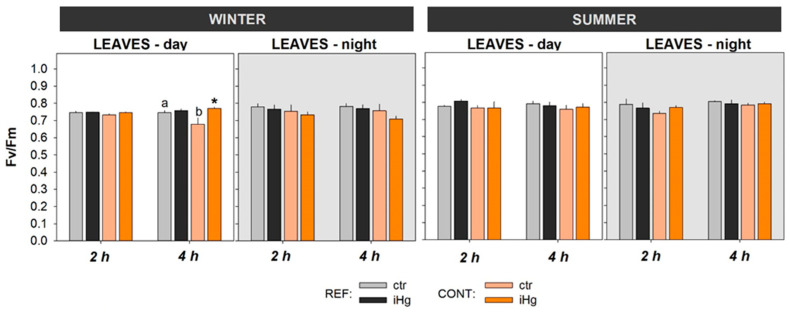
Photosynthetic activity in the leaves of *Halimione portulacoides* collected/exposed in the winter and in the summer, depicting the efficiency of photosystem II (Fv/Fm). Plant specimens were collected from two different estuarine sites—minimally contaminated (reference; REF) and historically contaminated with Hg (CONT)—and laboratory-exposed to 0.45 µg L^−1^ inorganic Hg (iHg) for 2 and 4 h during the day (left-hand white panels) or the night (right-hand gray panels). For each plant organ and provenance, values (bars represent the mean of three replicated measurements and the error bars represent the standard error) concern unexposed (ctr) and exposed (iHg) groups. Diurnal and nocturnal effects of site/origin (REF vs. CONT) and treatment (ctr vs. iHg) in terms of statistical differences are given based on Tukey tests following each omnibus two-way ANOVA, as summarized in Table S4 (*p* < 0.05). * Significantly different from ctr within the same site/origin and exposure length; different letters represent significant differences between different sites/origins within the same treatment.

The photosynthetic activity of the experimental plants was generally similar between seasons and between treatments within the same season (Figure 4). Still, it should be noticed that exposure to iHg drove a significant increase in Fv/Fm records following a 4 h diurnal exposure in the winter during daytime (Figure 5 and Table S4). In addition, Fv/Fm records depicted significantly lower levels in unexposed plants sourced from the CONT site compared to the REF site in the winter during the diurnal experiment (4 h).

## 4. Discussion

The decision to approach a (very) short-term context in the present study relied on the understanding that it can provide critical information about iHg potential for acute toxicity, shedding light on the mechanisms through which this metal form may harm *H. portulacoides*, and helping also to identify specific targets or pathways that make it vulnerable (or resistant). It should be noted that this will not capture the full range of potential effects and how they evolve over time, and thus, a comprehensive understanding of the dynamic nature of saltmarsh plant toxicological responses to iHg requires long-term toxicity studies to complement these findings. Thus, the present research was run in parallel with a long-term study whose results will be published shortly, allowing their integration with those now presented. Nevertheless, Cabrita et al. [5] preceded the current research, demonstrating Hg isotope accumulation in a comparable short-term (1 to 4 h) waterborne exposure.

### 4.1. Mercury Accumulation and Its Modulation by Environmental Factors and Ecological Traits

A poor accumulation of iHg was noticed, but a significant enrichment in Hg in the roots of CONT plants exposed to iHg for 2 h only, under hydroponic conditions simulating winter and daylight, indicates that this Hg form was available for root uptake. This finding also indicates that iHg uptake by the saltmarsh plant *H. portulacoides* is a short-term process (within a couple of hours), even under realistic waterborne exposure levels (0.45 μg L^−1^). Comparable concentrations were found in the water of contaminated systems [6]. Although not statistically different, higher Hg accumulation levels were also found in the roots of plants from the CONT site that were exposed for 4 h (mean levels in exposed and control plants were 0.15 and 0.09 μg g^−1^, respectively). The uptake of Hg by saltmarsh plants has been widely described, but mostly in field studies that reported the accumulation of this element in the roots [3,11,29], while short-term studies are scarce. Cabrita et al. [5] pioneered in this context, with plants from another system, by describing for the first time the uptake and transport of Hg isotopes in *H. portulacoides* grown under hydroponic conditions. The findings of Cabrita et al. [5] described a significant accumulation of Hg forms within a few hours of exposure (1–4 h). Trace metal uptake by plants from the surrounding environment follows the same transport pathways as the ones used by micronutrient metal ions [30]. Hg cations (as Hg(II)) have a high affinity for sulphydryl groups, facilitating their uptake through sequestration into cysteine-rich peptides, namely metallothioneins and phytochelatins, by binding to organic sulfur groups [5]. The main pathway of Hg(II) entering the roots was recently confirmed, consisting of its binding to lower molecular organic matter (as cysteine) [31].

Although the uptake of iHg in the roots of plants from the contaminated saltmarsh was found in winter during daylight exposure, no significant enhancements in Hg levels in the stems and leaves were recorded. A different pattern was described by Cabrita et al. [5] upon *H. portulacoides’* short-term exposure to Hg isotopes, demonstrating Hg translocation to the stems and leaves within a few hours. Environmentally realistic levels were used in the current experiment (0.45 μg L^−1^) with *H. portulacoides*, which combined with the short-term exposure could explain the lack of a detectable translocation for the aboveground organs. Any findings showing discrepancy with those of Cabrita et al. [5] could be related to exposure levels, which were indeed doubled in that study for the iHg isotope (1.05 μg L^−1^). The summer findings follow in the same direction as those of the winter-simulated conditions, with uptake in the roots of plants from the most contaminated area and poor translocation to the stems and leaves. Mercury accumulation was found in the roots of plants from the CONT site upon iHg exposure for 4 h under darkness. Despite this, no significant enhancements in Hg in the stems were found, suggesting poor translocation to the aboveground organs, as recorded in winter conditions.

Previous studies have found that light at proper intensity, spectral quality, and photoperiod can fuel plant growth and increase the efficiency of soil nutrient absorption [32]. Thus, a role of light on trace element uptake in saltmarsh plants could be speculated, as the same uptake pathways are used for micronutrient metal ions and trace elements [30]. Despite this, the current results on *H. portulacoides* have not evidenced the role of light on the Hg(II) uptake.

The current experiment also simulated winter and summer conditions regarding temperature and photoperiod. Different uptake patterns were found for *H. portulacoides* exposed in winter- and summer-simulated conditions, but current data do not support conclusions on the season that is more favorable to iHg uptake under controlled laboratorial conditions. Despite this, winter plants from both saltmarshes had, in general, higher levels of Hg than those collected in summer. This is probably related to a seasonal pattern of Hg accumulation in Aveiro lagoon saltmarshes. In fact, seasonal variations in Hg accumulation in *H. portulacoides* at Aveiro lagoon were investigated, documenting slightly higher levels in the winter than the summer, particularly in the stems and leaves [11]. Differences between winter and summer patterns may also be due to a net higher accumulation associated with the slower growth of plants occurring during the winter months.

Inorganic Hg uptake was only found in the roots of plants from the contaminated saltmarsh, while plants from the REF area did not have an efficient uptake. This points out the role of *H. portulacoides’* background in terms of contamination on the uptake of iHg. CONT and REF plants were subjected to distinct contamination levels, as pointed out by the total levels of Hg found in the surface sediments collected from those sites during the same field winter campaign, with values ranging from 1.87 to 2.98 μg g^−1^ at the REF site and 11 to 22 μg g^−1^ at the CONT site (Canário, in preparation). Slightly lower values were found in the surface sediments of those sites in the summer of 2021, with levels ranging as follows: 0.08–14.2 μg g^−1^ at the REF site and 4.8–18.6 μg g^−1^ at the CONT site (Canário, in preparation). Although the REF site was not a pristine site for Hg, this study aimed to compare the responses of the same saltmarsh plant species (*Halimione portulacoides*) with distinct contamination backgrounds to Hg(II). This would imply that the plants need to be collected from the same ecosystem. The selected reference site was a saltmarsh with much lower levels of Hg than the contaminated site, and distant enough from the hotspot. The selection of an alternative sampling site at an increased distance from the Laranjo area would probably imply the use of plants with different physiological characteristics than those collected at the hotspot site. This would probably prevent interpretations on adaptative responses to Hg(II), as was the aim in this study. Indeed, the distinct provenance of the plants seems to promote divergent Hg accumulation levels, as widely reported in the field [11]. Despite this, the two months of acclimation were enough to promote a reset on those differences with the growth of new radicular tissue. In fact, average levels in the roots of the REF and CONT plants immediately before exposure in winter-simulated conditions were 0.045 ± 0.020 μg g^−1^ and 0.046 ± 0.008 μg g^−1^, respectively. However, only the roots of the CONT plants had an efficient uptake of iHg, as demonstrated by the significant enhancement in accumulation levels in comparison to the controls (after 2 h of exposure in daylight/winter conditions and after 4 h of exposure under darkness/summer conditions), supporting the discussion on the role of plant contamination background history on iHg uptake, which represents a new analytical perspective of this process.

### 4.2. Organ-Specific Oxidative Stress Responses to iHg as a Function of Light Exposure, Season, and Exposure History

Redox homeostasis has been stated as the “Golden Mean” of healthy living [33]. This was the foundational idea for the questions raised in the present study and for the interpretation of the results obtained, as redox homeostasis is regarded as a structuring guideline for *H. portulacoides*’ responses to the challenges (eliminated them, preventing damage) posed by iHg, therefore determining its toxicodynamics.

The root and leaf responses currently detected in terms of antioxidant modulation make it difficult to establish variation profiles as a function of iHg exposure (and internal concentrations detected) and exposure duration. While simplifying explanations is attractive and (misleadingly) more effective in science, it often does not reflect an approximation to reality; the framework under study is a paradigmatic example of this. Consequently, it must be brought to the fore that the maintenance of a physiological redox steady state through the intervention of the antioxidant system depends on signaling pathways (e.g., electrophiles) and the signal transduction that takes place through the fine adjustment of the rheostat, rather than by the flipping of an on–off switch, involving rapid feedback reactions [33].

Anyway, it was discernible that, in winter, the leaves of plants sourced from the REF site, when showing alterations in the assessed enzymatic antioxidants, displayed an activity increment as a pattern of response to iHg exposure (with a single exception for SOD following daytime exposure for 4 h). In the leaves of plants from the CONT site in the winter, as well as in the leaves of plants from both origins in the summer, the antioxidants were much less responsive and showed an irregularity in the direction of variation. Highlighting an organ-specific profile of response in the roots, the alterations always reflected antioxidant activity decreases, with particular emphasis in the CONT groups in both seasons and in the REF groups in the winter.

Increases in antioxidant activities are easily explained as a self-correcting physiological response to iHg challenges. Differently, a reduction in these activities could suggest either an inhibition or a decreased expression/synthesis of the antioxidant enzymes. The hypothesis of activity inhibition would represent a clear sign of toxicity, with the consequence of increased risk of oxidative damage, which was not corroborated by the LPO results. Thus, the second explanation gains plausibility and reflects the operation of efficient feedback pathways on antioxidant modulation, keeping redox homeostasis in mind. This instantaneous picture of part of the antioxidant system evidenced a low pro-oxidative pressure in *H. portulacoides* challenged by iHg, allowing endogenous resources to be saved via a lower expression/synthesis of enzymatic antioxidants. This can be regarded as a new homeostatic condition, resulting from an overcompensation response to a mild challenge within a limited time period that allows the re-establishment and appropriate allocation of cell resources, as proposed by Calabrese and Baldwin [34].

Halophytes have developed a suite of traits, besides salt tolerance mechanisms, that give them competitive advantages, including reinforced antioxidant defenses [35]. Thus, it can be hypothesized that, given the high constitutive levels of antioxidants (enzymatic and non-enzymatic) in *H. portulacoides*, the responsiveness of antioxidant enzymes may be reduced, respecting the principle of proportionality of response to the oxidative insult. Biological systems must be able to react to challenges at a reasonable level [33]. In this framework, and paying particular attention to the results in the winter experiment for the leaves, plants from the CONT site seemed to have higher thresholds to the induction of antioxidant enzymes compared with those from the REF site. This denotes that the former subset is better prepared to cope with iHg redox pressure.

The lipid peroxidative damage results unveiled the most impressive identification of uncommon attributes of *H. portulacoides*, since both subsets of this halophyte population showed the capability of preventing peroxidative damage in both organs. This means that the species is properly equipped with an antioxidant shield that ensures that the boundary of the physiological redox steady state is not breached in a short timescale. In the same way, the detection of lower levels of peroxidative damage in the leaves following plant exposure to iHg is even more remarkable, and is in accordance with the poor translocation of Hg(II) from the roots to the leaves. In the winter, this profile of response was restricted to plants from the CONT site exposed during daytime (for both exposure lengths), which agrees with the suggestion presented above towards a better protection of this population subset. Nonetheless, it must be pointed out that this (apparent) beneficial response in the CONT groups cannot be dissociated from the fact that they had higher LPO levels in the unexposed groups (ctr) when compared to the REF groups.

In the summer experiment, this pattern of LPO response was exhibited by the leaves of plants from both provenances (REF at daytime; REF and CONT at nighttime) following the shorter exposure.

The biological meaning of this paradoxical effect and its assumption as a toxicologically based mechanistic strategy is not consensual, but, in our opinion, it fits the concept of “overcompensation hormesis” (OCSH). According to Calabrese and Baldwin [34], OCSH is “an adaptive response to low levels of stress or damage resulting in enhanced fitness for some physiological systems for finite periods and, under specific defined circumstances (…)”. It can be regarded as a modest overcompensation to a disruption in homeostasis, generating re-establishment and setting up a process of adaptive nature [34]. In light of this conceptualization, the adjustment features exhibited by *H. portulacoides* fit into the paradigm of resistance/adaptation, involving the establishment new homeostatic settings and the corresponding phenotypic shift towards a permanent modification of a function.

The analysis of the literature on this topic makes clear the lack of research on the influence of abiotic factors, such as temperature and light, as well biological traits depending on Hg exposure history, highlighting the novelty of the present study. In general, no clear patterns could be discerned on the effects of light, or of season, on the iHg toxicodynamics in *H. portulacoides* in this short-term exposure experiment. However, a sole exception was found for CAT activity, which was much higher in the summer experiment than in the winter. This finding is in line with the investigation of seasonal effect on the antioxidant activity in *Brassica* vegetables, demonstrating the influence of season on the concentration of the bioactive components of plants and antioxidant activity [36]. Regarding the historic contamination background, the CONT plants seemed to have higher thresholds for the induction of antioxidant enzymes compared to those from the REF site (as evidenced by leaf results), denoting that the former subset is better prepared to cope with iHg redox pressure, as previously discussed. Peroxidative damage findings pointed in the same direction, as the leaves of the CONT plants exposed to iHg had remarkably lower levels of LPO. This assumption, based on antioxidant protection and peroxidative damage, corroborates with that suggested by the Hg accumulation data, pointing out CONT plants as more prone to the uptake of iHg.

It was also demonstrated that biochemical markers related to oxidative stress are highly sensitive for translating cellular disturbances/adjustments. In fact, effects at this level were detected in a (very) short timescale, even when significant increments of iHg accumulation were undetectable in specific tissues by conventional quantification methods. The early interaction of iHg with cellular components can disrupt cellular functions or activate biochemical pathways, initiating adaptive processes before a substantial amount of metal accumulates intracellularly.

### 4.3. Modulation of Photosynthetic Efficiency by iHg Exposure

It has been demonstrated that the photosynthesis process is primarily affected under Hg treatment, hindering the functionality of PS II in different plant species [37]. Though not so well-stablished, this vulnerability was also described in halophytes, where iHg deregulated several proteins that take part in the process of photosynthesis [38].

In contrast, the present observations revealed that this critical physiological process was largely unaffected in the experimental plants following exposure to iHg. This reinforces the interpretation made above on *H. portulacoides*’ capacity to maintain a physiological balance. Furthermore, in the winter exposure (4 h) during daytime, the plants from the CONT site displayed an increase in photosynthetic performance, which coincided in time with a reduction in LPO levels. Taking these observations together, these two effects corroborate the assumption that an OCSH strategy has a determining (dampening) impact on plant interactions with iHg.

### 4.4. Overall Interpretations and Findings Extrapolation

The adaptive capacity of *H. portulacoides* to mitigate the immediate effects of iHg through rapid biochemical and enzymatic responses was demonstrated, also highlighting the sensitivity and accuracy of the respective analytical methodologies, which displayed a better performance compared to those currently adopted to detect Hg.

Homeostasis must be interpreted as a looked-for condition by the organism as a whole (or even by the population), so that, in theoretical terms, it cannot be excluded that a punctual imbalance in a given part of the body may occur, provided that the survival and efficiency of the whole is assured. Nonetheless, both the roots and leaves of *H. portulacoides* were able to prevent, regionally, a stable alteration in redox homeostasis, that is to say, they were able to avoid oxidative stress. Though only a short period was addressed, this sustains the assumption that the plant as a whole did not suffer critical variations in its health condition, which, interestingly, was confirmed by the current photobiology data.

At this point, it is important to reiterate the attempt to unravel whether the processes underlying the successful response of *H. portulacoides* to iHg reflect a phenomenon of tolerance or resistance/adaptation. Considering the long period of acclimatization to which the plants were subjected to allow the resetting of mechanisms and respective signaling pathways (as corroborated, in general, by the data on tHg accumulation), as well as the exhibition of an almost comparable capacity in the two subsets of *H. portulacoides* populations (REF and CONT), the most plausible hypothesis is that of a resistance/adaptation phenomenon. Nevertheless, this assumption needs further validation. It is very much unplausible that the inconsistency found between accumulation levels of Hg and biological effects could be related to the accumulation of other trace elements in the plant’s tissues. This is because Hg is the most relevant/preponderant trace element in the abiotic matrices (water and sediment) of the CONT site. Moreover, the plants were allowed to acclimatize for 2 months prior to exposure, supporting the growth of new radicular tissue and the mobilization/elimination of Hg from the aboveground organs, and the plants were exposed to Hg(II) only.

The features described, likely encompassing toxicokinetic and toxicodynamic adjustments, should be interpreted as part of a complex net of mechanisms operating in *H. portulacoides*, probably depending on a genetic plasticity, that allows this species to smooth environmental shocks, including exposure to iHg in pore water.

Halophytes are the foundation of saltmarsh ecosystems, and thus, alterations in their physiology due to Hg exposure may have an impact on higher levels of ecological organization. Hence, it can be hypothesized that the individual feature described above has a favorable ecological impact, emerging from the species to colonize Hg-impacted areas, cover contaminated sediments, and thus, protect the ecosystem against erosion.

## 5. Conclusions

Overall, the outputs of the present study allow the following conclusions:

(i) Both subsets of the *H. portulacoides* population were able to maintain redox homeostasis and photosynthesis efficiency under a very-short-term exposure to a realistic concentration of iHg. However, plants from the site impacted by Hg (CONT) were revealed to be better suited to cope with this environmental challenge, probably taking advantage of a strategy frameable in an overcompensation hormesis model.

(ii) The study reinforces the knowledge on the (genetic/physiologic) plasticity of *H. portulacoides* and the ecological/biological attributes that determine the success of this species in saltmarshes historically contaminated by Hg.

(iii) No clear effect of the factors of light and season was discerned on iHg uptake and subsequent *H. portulacoides* response.

(iv) An inconsistency in Hg accumulation patterns was perceived (with no evidence of translocation to the stems and leaves), explained by a combination of factors related to exposure duration and the related method detection limits; this nods to the higher suitability of biochemical assay-based approaches in this timescale.

## Data Availability

The data presented in this study are available in the article and Supplementary Materials.

## Supplementary Materials

The following supporting information can be downloaded at: https://www.mdpi.com/article/10.3390/toxics12030211/s1. Table S1: Two-way ANOVA summary regarding the inspection of significant effects of Hg concentration, site of origin of tested plants, and their interactions, regarding iHg concentrations in roots, stems or leaves measured following 2 or 4 h of exposure in experiments held during the day or night; Table S2: Two-way ANOVA summary regarding the inspection of significant effects of iHg concentration, site of origin of tested plants, and their interactions, in leaves, for different oxidative stress and damage endpoints measured following 2 or 4 h of exposure during experiments held during the day or night; Table S3: Two-way ANOVA summary regarding the inspection of significant effects of iHg concentration, site of origin of tested plants, and their interactions, in roots, for different oxidative stress and damage endpoints measured following 2 or 4 h of exposure during experiments held during the day or night; Table S4: Two-way ANOVA summary regarding the inspection of significant effects of iHg concentration, site of origin of tested plants, and their interactions, for Fv/Fm measured following 2 or 4 h of exposure in experiments held during the day or night. ANOVA assumptions of normality and homocedasticity were met in all analyses, as confirmed by the Shapiro-Wilk test (*p* > 0.05) and the Levene’s test (*p* > 0.05), respectively.

## References

[1] Gedan K.B., Silliman B.R., Bertness M.D. (2009). Centuries of Human-Driven Change in Salt Marsh Ecosystems. Ann. Rev. Mar. Sci..

[2] Roe R.A.L., Yu R.M.K., Rahman M.M., MacFarlane G.R. (2021). Towards Adverse Outcome Pathways for Metals in Saltmarsh Ecosystems—A Review. J. Hazard. Mater..

[3] Canário J., Vale C., Poissant L., Nogueira M., Pilote M., Branco V. (2010). Mercury in Sediments and Vegetation in a Moderately Contaminated Salt Marsh (Tagus Estuary, Portugal). J. Environ. Sci..

[4] Canário J., Poissant L., Pilote M., Caetano M., Hintelmann H., O’Driscoll N.J. (2017). Salt-Marsh Plants as Potential Sources of Hg0 into the Atmosphere. Atmos. Environ..

[5] Cabrita M.T., Duarte B., Cesário R., Mendes R., Hintelmann H., Eckey K., Dimock B., Caçador I., Canário J. (2019). Mercury Mobility and Effects in the Salt-Marsh Plant *Halimione portulacoides*: Uptake, Transport, and Toxicity and Tolerance Mechanisms. Sci. Total Environ..

[6] Pereira P., Raimundo J., Araújo O., Canário J., Almeida A., Pacheco M. (2014). Fish Eyes and Brain as Primary Targets for Mercury Accumulation—A New Insight on Environmental Risk Assessment. Sci. Total Environ..

[7] Natasha N., Shahid M., Khalid S., Bibi I., Bundschuh J., Khan Niazi N., Dumat C. (2020). A Critical Review of Mercury Speciation, Bioavailability, Toxicity and Detoxification in Soil-Plant Environment: Ecotoxicology and Health Risk Assessment. Sci. Total Environ..

[8] Figueira E., Matos D., Cardoso P., Pires A., Fernandes C., Tauler R., Bedia C. (2023). A Biochemical and Lipidomic Approach to Perceive *Halimione portulacoides* (L.) Response to Mercury: An Environmental Perspective. Mar. Pollut. Bull..

[9] Anjum N.A., Israr M., Duarte A.C., Pereira M.E., Ahmad I. (2014). *Halimione portulacoides* (L.) Physiological/Biochemical Characterization for Its Adaptive Responses to Environmental Mercury Exposure. Environ. Res..

[10] Maury-Brachet R., Ribeyre F., Boudou A. (1990). Actions and Interactions of Temperature and Photoperiod on Mercury Accumulation by Elodea Densa from Sediment Source. Ecotoxicol. Environ. Saf..

[11] Anjum N.A., Ahmad I., Válega M., Pacheco M., Figueira E., Duarte A.C., Pereira E. (2011). Impact of Seasonal Fluctuations on the Sediment-Mercury, Its Accumulation and Partitioning in Halimione Portulacoides and Juncus Maritimus Collected from Ria de Aveiro Coastal Lagoon (Portugal). Water Air Soil Pollut..

[12] Liu X., Wu H., Ji C., Wei L., Zhao J., Yu J. (2013). An Integrated Proteomic and Metabolomic Study on the Chronic Effects of Mercury in Suaeda Salsa under an Environmentally Relevant Salinity. PLoS ONE.

[13] Santos D., Duarte B., Caçador I. (2014). Unveiling Zn Hyperaccumulation in Juncus Acutus: Implications on the Electronic Energy Fluxes and on Oxidative Stress with Emphasis on Non-Functional Zn-Chlorophylls. J. Photochem. Photobiol. B Biol..

[14] Anjum N.A., Duarte B., Caçador I., Sleimi N., Duarte A.C., Pereira E. (2016). Biophysical and Biochemical Markers of Metal/Metalloid-Impacts in Salt Marsh Halophytes and Their Implications. Front. Environ. Sci..

[15] Canário J., Caetano M., Vale C., Cesário R. (2007). Evidence for Elevated Production of Methylmercury in Salt Marshes. Environ. Sci. Technol..

[16] Coelho J.P., Pereira M.E., Duarte A., Pardal M.A. (2005). Macroalgae Response to a Mercury Contamination Gradient in a Temperate Coastal Lagoon (Ria de Aveiro, Portugal). Estuar. Coast. Shelf Sci..

[17] Ribeiro B.C. (2022). Trace Element Contamination and Distribution in Surface Sediments in Ria de Aveiro. Master’s Dissertation.

[18] Válega M., Lillebø A.I., Caçador I., Pereira M.E., Duarte A.C., Pardal M.A. (2008). Mercury Mobility in a Salt Marsh Colonised by Halimione Portulacoides. Chemosphere.

[19] Silva H., Caldeira G., Freitas H. (2007). Salicornia Ramosissima Population Dynamics and Tolerance of Salinity. Ecol. Res..

[20] Souri Z., Cardoso A.A., Da-Silva C.J., de Oliveira L.M., Dari B., Sihi D., Karimi N. (2019). Heavy Metals and Photosynthesis: Recent Developments. Photosynthesis, Productivity and Environmental Stress.

[21] Miller J.N., Miller J.C. (2010). Statistics and Chemometrics for Analytical Chemistry.

[22] Duarte B., Santos D., Marques J.C., Caçador I. (2015). Impact of Heat and Cold Events on the Energetic Metabolism of the C3 Halophyte Halimione Portulacoides. Estuar. Coast. Shelf Sci..

[23] Teranishi Y., Tanaka A., Osumi M., Fukui S. (1974). Catalase Activities of Hydrocarbon-Utilizing Candida Yeasts. Agric. Biol. Chem..

[24] Tiryakioglu M., Eker S., Ozkutlu F., Husted S., Cakmak I. (2006). Antioxidant Defense System and Cadmium Uptake in Barley Genotypes Differing in Cadmium Tolerance. J. Trace Elem. Med. Biol..

[25] Bradford M.M. (1976). A Rapid and Sensitive Method for the Quantitation of Microgram Quantities of Protein Utilizing the Principle of Protein-Dye Binding. Anal. Biochem..

[26] Anjum N.A., Ahmad I., Rodrigues S.M., Henriques B., Cruz N., Coelho C., Pacheco M., Duarte A.C., Pereira E. (2013). Eriophorum Angustifolium and Lolium Perenne Metabolic Adaptations to Metals- and Metalloids-Induced Anomalies in the Vicinity of a Chemical Industrial Complex. Environ. Sci. Pollut. Res..

[27] Dhindsa R.S., Plumb-dhindsa P., Thorpe T.A. (1981). Leaf Senescence: Correlated with Increased Levels of Membrane Permeability and Lipid Peroxidation, and Decreased Levels of Superoxide Dismutase and Catalase. J. Exp. Bot..

[28] Serôdio J., Vieira S., Cruz S. (2008). Photosynthetic Activity, Photoprotection and Photoinhibition in Intertidal Microphytobenthos as Studied in Situ Using Variable Chlorophyll Fluorescence. Cont. Shelf Res..

[29] Castro R., Pereira S., Lima A., Corticeiro S., Válega M., Pereira E., Duarte A., Figueira E. (2009). Accumulation, Distribution and Cellular Partitioning of Mercury in Several Halophytes of a Contaminated Salt Marsh. Chemosphere.

[30] Patra M., Bhowmik N., Bandopadhyay B., Sharma A. (2004). Comparison of Mercury, Lead and Arsenic with Respect to Genotoxic Effects on Plant Systems and the Development of Genetic Tolerance. Environ. Exp. Bot..

[31] Yuan W., Wang X., Lin C.-J., Wu F., Luo K., Zhang H., Lu Z., Feng X. (2022). Mercury Uptake, Accumulation, and Translocation in Roots of Subtropical Forest: Implications of Global Mercury Budget. Environ. Sci. Technol..

[32] Luo J., Cao M., Zhang C., Wu J., Gu X.W.S. (2020). The Influence of Light Combination on the Physicochemical Characteristics and Enzymatic Activity of Soil with Multi-Metal Pollution in Phytoremediation. J. Hazard. Mater..

[33] Ursini F., Maiorino M., Forman H.J. (2016). Redox Homeostasis: The Golden Mean of Healthy Living. Redox Biol..

[34] Calabrese E.J., Baldwin L.A. (2002). Defining Hormesis. Hum. Exp. Toxicol..

[35] Lima A.R., Castañeda-Loaiza V., Salazar M., Nunes C., Quintas C., Gama F., Pestana M., Correia P.J., Santos T., Varela J. (2020). Influence of Cultivation Salinity in the Nutritional Composition, Antioxidant Capacity and Microbial Quality of Salicornia Ramosissima Commercially Produced in Soilless Systems. Food Chem..

[36] Aires A., Fernandes C., Carvalho R., Bennett R.N., Saavedra M.J., Rosa E.A.S. (2011). Seasonal Effects on Bioactive Compounds and Antioxidant Capacity of Six Economically Important Brassica Vegetables. Molecules.

[37] Singh H., Kumar D., Soni V. (2023). Impact of Mercury on Photosynthetic Performance of Lemna Minor: A Chlorophyll Fluorescence Analysis. Sci. Rep..

[38] Liu T., Chen Q., Zhang L., Liu X., Liu C. (2021). The Toxicity of Selenium and Mercury in Suaeda Salsa after 7-Days Exposure. Comp. Biochem. Physiol. Part C Toxicol. Pharmacol..

