# Supplementary material for: Redox Homeostasis Disclosed in the Saltmarsh Plant Halimione portulacoides upon Short Waterborne Exposure to Inorganic Mercury
Source: Toxics. 2024 Mar 12;12(3):211. doi: 10.3390/toxics12030211 (PMC10974591; doi:10.3390/toxics12030211)
Supplement: Supplementary file 1 [file toxics-12-00211-s001.zip › toxics-2824963-supplementary.pdf]

Supplementary Material

Table S1. Two-way ANOVA summary regarding the inspection of significant effects of Hg concentration, site of origin of tested plants, and their interactions, regarding iHg concentrations in roots, stems or leaves measured following 2 or 4 h of exposure in experiments held during the day or night.

**WINTER**																		
	**DAY . t = 2 h**				**DAY . t = 4 h**				**NIGHT . t = 2 h**					**NIGHT . t = 4 h**				
Source of variation	df	MS	F	P	df	MS	F	P	df	MS	F	P		df	MS	F	P	
ROOTS																		
[iHg]	1	0.0761	39.323	**<0.001**	1	0.0035	1.158	0.313	1	0.0057	0.786	0.401		1	0.0024	2.607	0.150	
Site	1	0.0386	19.941	**0.002**	1	0.0130	4.287	0.072	1	0.0084	1.166	0.312	*	1	1.45e-4	0.158	0.730	
[iHg]*Site	1	0.0452	23.390	**0.001**	1	0.0015	0.507	0.497	1	0.0062	0.864	0.380		1	0.0031	3.412	0.107	
Error	8	0.0155			8	0.0030			8	0.0072				7	9.13e-4			
STEMS																		
[iHg]	1	0.0068	15.92	**0.004**	1	0.0023	2.672	0.141	1	0.0013	2.092	0.186		1	9.99e-5	0.113	0.745	
Site	1	0.0027	6.210	0.037	1	0.0073	8.455	**0.020**	1	0.0114	19.016	**0.002**		1	0.0135	15.32	**0.004**	
[iHg]*Site	1	8.07e-5	0.188	0.676	1	1.80e-6	0.002	0.965	1	2.17e-4	0.362	0.564		1	0.0020	2.289	0.169	
Error	8	4.30e-4			8	8.63e-4			8	6.00e-4				8	8.83e-4			
LEAVES																		
[iHg]	1	9.66e-5	0.028	0.871	1	0.0108	0.999	0.347	1	0.0089	1.492	0.257		1	8.43e-5	0.008	0.931	
Site	1	0.0074	2.152	0.181	1	0.0023	0.218	0.653	1	0.0046	0.770	0.406	×	1	0.0072	0.685	0.432	
[iHg]*Site	1	0.0068	1.980	0.197	1	0.0080	0.741	0.414	1	0.0098	1.644	0.236		1	0.0043	0.411	0.539	
Error	8	0.0035			8	0.0108			8	0.0060				8	0.0105			
**SUMMER**																		
	**DAY . t = 2 h**				**DAY . t = 4 h**				**NIGHT . t = 2 h**					**NIGHT . t = 4 h**				
Source of variation	df	MS	F	P	df	MS	F	P	df	MS	F	P		df	MS	F	P	
ROOTS																		
[iHg]	1	0.0012	1.279	0.291	1	1.95e-5	0.078	0.787	1	0.0018	1.949	0.200		1	0.0034	7.327	**0.030**	
Site	1	0.0097	10.27	**0.013**	1	0.0038	15.23	**0.005**	1	0.0235	26.13	**<0.001**		1	0.0064	13.59	**0.008**	×
[iHg]*Site	1	8.67e-6	0.009	0.926	1	5.94e-5	0.237	0.640	1	4.69e-4	0.521	0.491		1	0.0012	2.565	0.153	
Error	8	9.42e-4			8	2.51e-4			8	8.99e-4				7	4.69e-4			
STEMS																		
[iHg]	1	2.59e-4	4.278	0.072	1	8.16e-5	0.829	0.389	1	2.08e-6	0.010	0.923		1	1.33e-6	0.0185	0.895	
Site	1	2.05e-5	0.340	0.576	1	4.41e-7	0.0045	0.948	1	8.33e-8	0.0004	0.985		1	0.00154	21.358	**0.002**	
[iHg]*Site	1	9.67e-4	15.994	**0.004**	1	1.82e-4	1.846	0.211	1	6.75e-6	0.0325	0.861		1	1.47e-4	2.037	0.191	
Error	8	6.04e-5			8	9.85e-5			8	2.08e-4				8	7.22e-5			
LEAVES																		
[iHg]	1	0.0212	2.459	0.156	1	0.0148	5.322	0.050	1	0.0135	2.700	0.139		1	6.75e-6	6.38e-4	0.980	
Site	1	0.0299	3.478	0.099	1	1.04e-4	0.037	0.852	1	0.0237	4.723	0.061		1	0.0037	0.350	0.570	
[iHg]*Site	1	0.0024	0.280	0.611	1	0.0150	5.398	**0.049**	1	0.0023	0.453	0.520		1	8.17e-4	0.0772	0.788	
Error	8	0.0086			8	0.0028			8	0.0050				8	0.0106			

×Homocedasticity failed (Levene's test; p<0.05); *Normality failed (Shapiro-Wilk test; p>0.05). Parametric ANOVA was still run, considering that very few cases occurred and to keep consistency across the analysis.

Table S2. Two-way ANOVA summary regarding the inspection of significant effects of iHg concentration, site of origin of tested plants, and their interactions, in leaves, for different oxidative stress and damage endpoints measured following 2 or 4 h of exposure during experiments held during the day or night.

		DAY . t = 2 h				DAY . t = 4 h				NIGHT . t = 2 h				NIGHT . t = 4 h			
**WINTER**																	
	Source of variation	df	MS	F	P	df	MS	F	P	df	MS	F	P	df	MS	F	P
**CAT**	[iHg]	1	1.4967	0.15	0.709	1	0.174	0.0320	0.862	1	37.125	14.307	**0.009**	1	10.943	1.562	0.247
	Site	1	0.0143	0.00	0.971	1	0.0246	0.00452	0.948	1	1.003	0.386	0.557	1	6.796	0.970	0.354
	[iHg]*Site	1	358569	3.59	0.095	1	8.255	1.519	0.253	1	22.362	8.618	**0.026**	1	17.487	2.496	0.153
	Error	8	10.00			8	5.434			6	2.595			8	7.005		
**AP**	[iHg]	1	0.24412	0.41	0.538	1	1.934	1.968	0.198	1	0.0109	2.759	0.148	1	9.06e-4	0.379	0.555
	Site	1	0.07787	0.13	0.726	1	0.553	0.563	0.475	1	0.0225	0.571	0.478	1	0.0589	2.461	0.155
	[iHg]*Site	1	0.87106	1.48	0.259	1	1.744	1.775	0.219	1	9.06e-4	0.230	0.649	1	0.0769	3.210	0.111
	Error	8	0.59049			8	0.983			6	0.00394			8	0.0239		
**GP**	[iHg]	1	920.30	7.73	**0.027**	1	0.285	0.00451	0.948	1	1018.40	20.608	**0.002**	1	674.443	5.179	0.052
	Site	1	77.82	0.65	0.446	1	2.708	0.0427	0.841	1	39.326	0.796	0.398	1	95.023	0.730	0.418
	[iHg]*Site	1	95.86	0.80	0.399	1	705.500	11.135	**0.010**	1	608.857	12.321	**0.008**	1	349.093	2.680	0.140
	Error	7	119.10			8	63.358			8	49.418			8	130.238		
**SOD**	[iHg]	1	8.618	6.45	**0.044**	1	47.834	12.924	**0.011**	1	10.996	11.765	**0.011**	1	0.0467	0.0256	0.877
	Site	1	46.136	34.53	**0.001**	1	54.719	14.785	**0.009**	1	9.755	10.437	**0.014**	1	0.0288	0.0158	0.903
	[iHg]*Site	1	6.971	5.22	0.062	1	36.724	9.923	**0.020**	1	0.604	0.646	0.448	1	0.271	0.148	0.710
	Error	6	1.336			6	3.701			7	0.935			8	1.827		
**LPO**	[iHg]	1	1.79e-4	11.63	**0.009**	1	8.92e-5	25.625	**<0.001**	1	2.16e-5	1.028	0.340	1	1.17e-5	1.654	0.234
	Site	1	6.73e-6	0.437	0.527	1	1.17e-4	33.546	**<0.001**	1	4.11e-5	1.954	0.200	1	1.95e-5	2.754	0.136
	[iHg]*Site	1	7.13e-6	0.463	0.515	1	3.11e-5	8.932	**0.017**	1	2.22e-5	1.052	0.335	1	2.76e-5	3.888	0.084
	Error	8	1.54e-5			8	3.48e-6			8	2.11e-5			8	7.10e-6		
**SUMMER**																	
		DAY . t = 2 h				DAY . t = 4 h				NIGHT . t = 2 h				NIGHT . t = 4 h			
	Source of variation	df	MS	F	P	df	MS	F	P	df	MS	F	P	df	MS	F	P
**CAT**	[iHg]	1	2256.51	3.275	0.120	1	64.989	0.117	0.741	1	2233.06	8.815	**0.025**	1	7177.9	17.965	**0.003**
	Site	1	57.499	0.083	0.782	1	150.720	0.271	0.617	1	11954.6	47.189	**<0.001**	1	149.43	0.374	0.558
	[iHg]*Site	1	33.266	0.048	0.833	1	1425.55	2.561	0.148	1	781.344	3.084	0.130	1	2214.9	5.544	**0.046**
	Error	9	689.074			8	556.641			6	253.336			8	399.55		
**SOD**	[iHg]	1	233.539	2.828	0.136	1	0.0120	0.463	0.518	1	22.499	0.126	0.733	1	259.12	4.495	0.067
	Site	1	77.259	0.936	0.366	1	0.381	14.679	**0.006**	1	39.507	0.222	0.652	1	155.13	2.691	0.140
	[iHg]*Site	1	377.358	4.570	0.070	1	0.0094	0.363	0.566	1	23.541	0.132	0.727	1	17.821	0.309	0.593
	Error	7	82.572			7	0.363			7	178.340			8	57.651		
**LPO**	[iHg]	1	7.61e-5	18.71	**0.003**	1	3.42e-6	0.839	0.386	1	1.10e-4	23.393	**0.001**	1	1.62e-4	1.910	0.204
	Site	1	5.50e-6	1.354	0.278	1	1.83e-6	0.447	0.522	1	1.44e-6	0.308	0.594	1	1.06e-4	1.253	0.295
	[iHg]*Site	1	1.63e-5	4.008	0.080	1	1.01e-7	0.0248	0.879	1	5.76e-6	1.229	0.300	1	2.76e-4	3.260	0.109 ×
	Error	8	4.07e-6			8	4.08e-6			8	4.69e-6			8	8.46e-5		

×Homocedasticity failed (Levene's test; p<0.05), despite several attempts to transform the data – parametric ANOVA was still run, considering that very few cases occurred and to keep consistency across the analysis.

Table S3. Two-way ANOVA summary regarding the inspection of significant effects of iHg concentration, site of origin of tested plants, and their interactions, in roots, for different oxidative stress and damage endpoints measured following 2 or 4 h of exposure during experiments held during the day or night.

WINTER																					
		**DAY . t = 2 h**					**DAY . t = 4 h**					**NIGHT . t = 2 h**					**NIGHT . t = 4 h**				
	Source of variation	df	MS	F	P		df	MS	F	P		df	MS	F	P		df	MS	F	P	
**CAT**	[iHg]	1	2.394	0.861	0.381		1	1.527	0.858	0.381		1	0.752	0.303	0.597		1	9.332	3.776	0.093	
	Site	1	0.859	0.309	0.594		1	0.496	0.279	0.612		1	0.0573	0.0230	0.883		1	0.423	0.171	0.692	
	[iHg]*Site	1	0.589	0.212	0.658		1	0.162	0.0909	0.771		1	0.322	0.130	0.728		1	4.939	1.999	0.200	
	Error	8	2.781				8	1.780				8	2.485				7	2.471			
**AP**	[iHg]	1	0.461	1.815	0.215		1	0.0186	0.0874	0.776		1	0.170	0.591	0.464		1	2.452	15.417	**0.006**	
	Site	1	0.0910	0.359	0.566		1	0.343	1.611	0.245		1	0.276	0.958	0.356		1	0.517	3.251	0.114	×
	[iHg]*Site	1	0.286	1.128	0.319		1	0.666	3.127	0.120		1	0.548	1.907	0.205		1	0.103	0.645	0.448	
	Error	8	0.254				7	0.213				8	0.288				7	0.159			
**GP**	[iHg]	1	71.872	7.370	**0.026**		1	3.800	0.284	0.609		1	48.411	3.006	0.127		1	2.945	0.391	0.555	
	Site	1	2.369	0.243	0.635		1	11.000	0.821	0.391		1	59.562	3.698	0.096		1	0.121	0.0160	0.903	
	[iHg]*Site	1	17.942	1.840	0.212		1	1.213	0.0906	0.771		1	8.898	0.553	0.481		1	108.008	14.350	**0.009**	
	Error	8	9.751				8	13.391				7	16.104				6	7.527			
**SOD**	[iHg]	1	1.713	6.684	**0.032**		1	3.542	16.191	**0.004**		1	2.336	3.011	0.121		1	1.26e-4	0.0142	0.908	
	Site	1	0.0342	0.133	0.724		1	1.474	6.736	**0.032**		1	0.0546	0.0704	0.797		1	0.0601	6.785	**0.031**	
	[iHg]*Site	1	0.236	0.920	0.366		1	1.789	8.178	**0.021**		1	2.470	3.184	0.112		1	0.0635	7.180	**0.028**	
	Error	8	2.056				8	0.219				8	0.776				8	0.00885			
**LPO**	[iHg]	1	3.04e-5	2.690	0.140		1	8.80e-6	1.509	0.254		1	0.0424	1.674	0.232		1	9.00e-5	2.102	0.185	
	Site	1	2.10e-5	1.859	0.210		1	3.10e-5	5.320	**0.050**		1	0.292	11.505	**0.009**		1	3.34e-5	0.780	0.403	
	[iHg]*Site	1	4.86e-6	0.430	0.530		1	2.14e-6	0.368	0.561		1	1.17e-5	4.64e-4	0.983		1	3.08e-5	0.720	0.421	
	Error	8	1.13e-5				8	5.83e-6				8	0.0253				8	4.28e-5			
**SUMMER**																					
		**DAY . t = 2 h**					**DAY . t = 4 h**					**NIGHT . t = 2 h**					**NIGHT . t = 4 h**				
	Source of variation	df	MS	F	P		df	MS	F	P		df	MS	F	P		df	MS	F	P	
**CAT**	[iHg]	1	5715.07	6.787	0.040		1	491.693	1.507	0.274		1	3943.41	0.415	0.548		1	1143.51	0.162	0.701	
	Site	1	4966.28	5.898	0.051		1	1000.04	3.065	0.140		1	5349.30	0.563	0.487		1	70878.0	10.040	0.019	
	[iHg]*Site	1	9665.08	11.478	**0.015**		1	2293.36	7.028	**0.045**	×	1	60734.8	6.388	0.053		1	46848.6	6.636	**0.042**	×
	Error	6	842.029				8	326.317				5	9507.69				6	7059.80			
**SOD**	[iHg]	1	570.109	6.759	**0.035**		1	4.141	0.0388	0.849		1	170.383	0.708	0.425		1	398.301	1.381	0.278	
	Site	1	94.071	1.115	0.326		1	2.517	0.0236	0.882		1	20.123	0.0836	0.780		1	326.229	1.131	0.323	
	[iHg]*Site	1	242.130	2.871	0.134		1	37.843	0.355	0.540		1	1364.44	5.669	**0.044**	*	1	1232.15	4.273	0.078	
	Error	7	84.350				7	106.632				8	240.697				7	288.369			
**LPO**	[iHg]	1	2.29e-10	1.54e^{-5}	0.997		1	2.58e-4	7.838	**0.023**		1	3.74e-5	4.825	0.059		1	3.21e-5	7.353	0.027	
	Site	1	6.30e-5	4.231	0.074		1	5.20e-6	0.158	0.702	*	1	2.09e-6	0.269	0.618		1	7.78e-6	1.781	0.219	×
	[iHg]*Site	1	3.61e-7	0.0243	0.880		1	2.37e-9	7.18e-5	0.993		1	2.06e-6	0.265	0.620		1	7.30e-6	1.673	0.232	
	Error	8	1.49e-5				8	3.29e-5				8	7.75e-6				8	4.37e-6			

* Although the effects of the factor were significant in the omnibus ANOVA, post-hoc tests did not resolve any significant difference between sites or between treatments; ×Homocedasticity failed (Levene's test; $p<0.05$), despite several attempts to transform the data – parametric ANOVA was still run, considering that very few cases occurred and to keep consistency across the analysis.

Table S4. Two-way ANOVA summary regarding the inspection of significant effects of iHg concentration, site of origin of tested plants, and their interactions, for Fv/Fm measured following 2 or 4 h of exposure in experiments held during the day or night. ANOVA assumptions of normality and homocedasticity were met in all analyses, as confirmed by the Shapiro-Wilk test ($p > 0.05$) and the Levene's test ($p > 0.05$), respectively.

**WINTER**																
	**DAY . t = 2 h**				**DAY . t = 4 h**				**NIGHT . t = 2 h**				**NIGHT . t = 4 h**			
Source of variation	df	MS	F	P	df	MS	F	P	df	MS	F	P	df	MS	F	P
[iHg]	1	1.92e-4	2.118	0.184	1	0.00811	6.581	**0.033**	1	9.36e-4	0.476	0.510	1	0.00270	1.361	0.277
Site	1	1.92e-4	2.118	0.184	1	0.00224	1.818	0.214	1	0.00264	1.342	0.280	1	0.00546	2.753	0.136
[iHg]*Site	1	1.61e-4	1.779	0.219	1	0.00480	3.894	0.084	1	2.70e-5	0.0137	0.910	1	9.01e-4	0.454	0.519
Error	8	9.07e-5			8	0.00123			8	0.00197			8	0.00198		
**SUMMER**																
	**DAY . t = 2 h**				**DAY . t = 4 h**				**NIGHT . t = 2 h**				**NIGHT . t = 4 h**			
Source of variation	df	MS	F	P	df	MS	F	P	df	MS	F	P	df	MS	F	P
[iHg]	1	6.58e-4	0.992	0.353	1	2.02e-6	0.0016	0.969	1	1.20e-4	0.0736	0.793	1	4.11e-5	0.0784	0.787
Site	1	1.64e-3	2.473	0.160	1	1.21e-3	0.945	0.360	1	1.83e-3	1.120	0.321	1	3.98e-4	0.760	0.409
[iHg]*Site	1	6.66e-4	1.004	0.350	1	4.35e-4	0.340	0.576	1	2.42e-3	1.483	0.258	1	2.84e-4	0.542	0.483
Error	7	0.0047			8	0.0102			8	1.64e-3			8	5.24e-4		
